# Chiral Assembly Preferences and Directing Effects in Supramolecular Two-Component Organogels

**DOI:** 10.3390/gels4020031

**Published:** 2018-03-29

**Authors:** William Edwards, David K. Smith

**Affiliations:** Department of Chemistry, University of York, Heslington, York YO10 5DD, UK; William.Edwards@akzonobel.com

**Keywords:** chirality, organogel, peptide, self-assembly, two-component

## Abstract

The impact of chirality on the self-assembly of supramolecular gels is of considerable importance, as molecular-scale programming can be translated into nanostructuring and ultimately affect macroscopic performance. This paper explores the effect of chirality on the assembly of two-component gels comprised of a second-generation dendritic lysine peptide acid, containing three chiral centres, and an amine. This combination forms an acid–amine complex that assembles into nanofibres through peptide-peptide hydrogen bonds, leading to organogels. With achiral amines, a racemic mixture of l,l,l and d,d,d dendritic peptide acids surprisingly forms the best gels—more commonly, mixing enantiomers suppresses gelation. Thermodynamic studies demonstrate that depending on the amine, the greater stability of heterochiral gels can either be entropically or enthalpically driven. With amines possessing “*R*” chirality, the l,l,l peptide acid consistently forms more effective gels than its d,d,d analogue. Furthermore, in mixed gels, l,l,l sometimes imposes its assembly preference onto d,d,d. In summary, this paper demonstrates a rare example in which heterochiral gels are preferred, and also explores directing effects when each component in a two-component gel is chiral.

## 1. Introduction

Supramolecular gels are soft materials in which molecular-scale building blocks self-assemble into nanoscale architectures that form a sample-spanning “solid-like” network and hence immobilise a bulk solvent [1,2,3,4,5,6,7,8]. One of the most fascinating aspects of these materials is that “information” programmed-in at the molecular scale by organic synthesis can be translated up to the nanoscale via self-assembly, and ultimately control the macroscopic properties of the material. One key molecular architectural feature is chirality, which has been the subject of considerable investigation—the chiral organisation of groups responsible for molecular recognition can have a significant impact on self-assembly [9,10,11,12]. Given the importance of chiral assembly in biological soft matter systems, understanding such events is also of wider importance [13,14,15,16].

Multi-component gels have also been of considerable interest [17,18,19,20]. In a seminal review, Buerkle and Rowan [18] classified multi-component gels into different types: Type 1 multi-component gels require two (or more) molecules to form a complex, which is responsible for self-assembly and gelation [21,22,23]. Such systems are highly tunable and responsive—for example, one component can select another from a dynamic mixture [24,25,26,27,28]. Such systems also respond to stimulus and can adapt and evolve their composition in response to additives [29]. Type 2 multi-component gels have two different components, both independently capable of gelation [18]. The gelators either self-sort into their own networks [30,31,32,33,34,35,36,37,38,39,40,41,42,43], co-assemble into a combined network [44,45,46,47], or simply inhibit/prevent one another’s assembly. Type 3 multi-component gels just mix a second component into a preformed gel, which may, or may not directly interact with the gel fibres formed by the other component [48,49,50,51,52,53,54,55].

In terms of chirality in gels, mixing enantiomers, both of which are independently capable of gelation, has been of considerable interest [9,10,11,12]. This is a special case of a Type 2 multi-component gel. In the vast majority of cases, enantiopure gelators assemble better than their racemic analogues [56,57,58]—i.e., homochiral interactions are normally favoured over heterochiral ones. However, in a few exceptional cases, heterochiral racemates show enhanced gelation [59,60,61,62,63,64,65,66]. This can be assigned to a thermodynamic preference for heterochiral interactions—either at the molecular- or nanoscale. Chiral directing effects have also been reported in which a small amount of chiral additive can impose its preference onto an achiral system (a “sergeants and soldiers” effect), or a small excess of one enantiomer can impose its preference onto the other (a “majority rules” effect) [67,68,69,70,71,72,73,74].

**Figure 1:**
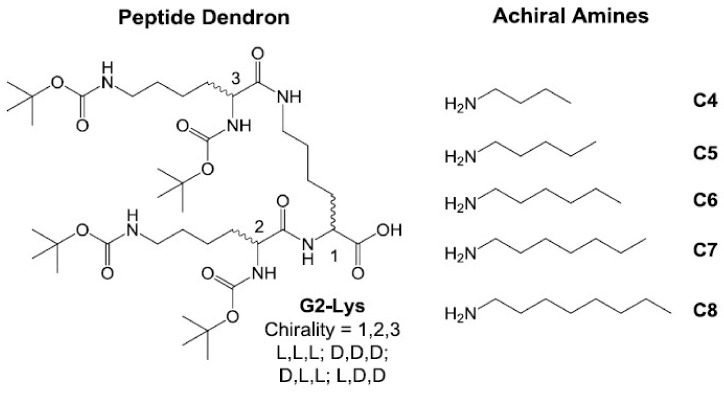
Two-component self-assembling system investigated in the first part of this paper.

In recent years, we have studied a versatile two-component Type 1 gelation system based on the complex formed between a lysine dendron with an acid group at the focal point (G2-Lys, Figure 1), and amines [29,75,76,77,78,79,80]. This complex hierarchically self-assembles via intermolecular peptide-peptide hydrogen bonds, with the amine modifying the solubility of the complex and encouraging the solid-like gel network to form. We have previously reported that the peptide dendron can “select” an amine from an enantiomeric mixture in order to form the thermodynamically favoured gel [81]. In this new study, we wanted to explore the effect of dendron chirality on self-assembly. The dendron is intimately involved in self-assembly, and we reasoned we might observe significant chirality effects. This study therefore challenges a Type 1 two-component gelation system with additional components also capable of forming gels (i.e., a Type 2 system) to provide an enhanced understanding of these multi-component gels.

## 2. Results and Discussion

### 2.1. Synthesis and Characterisation

The syntheses of chiral dendrons was achieved using standard methodologies based on peptide coupling and protecting group chemistry as previously described by us [82]. In particular, d,d,d-G2Lys (“D,D,D”) was synthesised for comparison with our enantiomeric standard dendron, l,l,l-G2Lys (“L,L,L”). Dendrons d,l,l-G2Lys (“D,L,L”) and l,d,d-G2Lys (“L,D,D”) were also synthesised—they have a diastereomeric relationship with L,L,L and an enantiomeric relationship with each other (Figure 1). We reasoned that this family of four stereoisomeric dendrons would allow us to probe the transfer of chiral information through intermolecular hydrogen bond pathways during self-assembly.

### 2.2. Investigation of Gel Formation with Simple Achiral Amines

The gelation of D,D,D and L,L,L was initially tested with achiral aliphatic amines (Figure 1) in toluene, and the gels were identical in each case in terms of thermal stability (*T*_gel_)—as expected for gels with an enantiomeric relationship. Similarly for D,L,L and L,D,D, the pairs of gels were identical (Table 1). However, these latter gels were different to those formed using D,D,D or L,L,L owing to the diastereomeric relationship. Indeed, different peptides formed optimal gels with different amines. It would appear that D,L,L (and L,D,D) is less sensitive to the amine chain length (except for C8), whereas L,L,L (and D,D,D) is clearly optimised to form gels with C6.

### 2.3. Effect of Enantiomeric Mixing on Gelation with Achiral Amines

**Figure 2:**
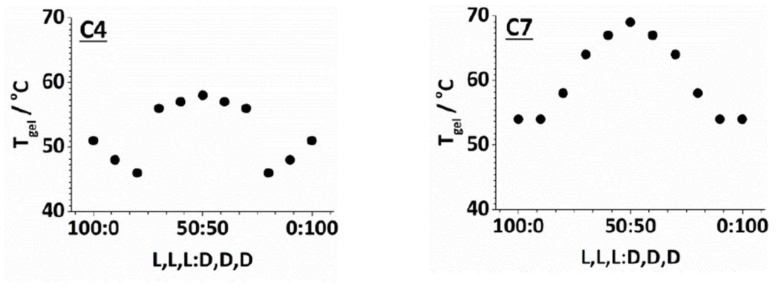
Typical examples of the effect of enantiomeric mixing on *T*_gel_, as measured by tube inversion. The concentration of amine in toluene is 10 mM, and the total concentration of dendron (D,D,D + L,L,L) is also 10 mM. The amines for which data are represented are C4 (**left**) and C7 (**right**). Data for all amines can be found in the Supplementary Material.

The effect of mixing peptide enantiomers was then investigated. We used simple, reproducible tube-inversion methodology to monitor the *T*_gel_ values of gels formed in toluene with 10 mM of aliphatic amine (C4–C8) and an overall 10 mM concentration of lysine dendron—composed of a varying ratio of L,L,L and D,D,D, such that all of the dendron should be able to bind to an amine and hence participate in gel formation. Typical examples of the effect of enantiomeric mixing are shown in Figure 2 (all data are presented in the Supplementary Material, Figure S1).

In all cases, the dependence of *T*_gel_ on the ratio of enantiomers was symmetrical around 50/50 L,L,L/D,D,D (i.e., the racemic gel). This reflects the enantiomeric relationship of the complexes. Further, in all cases, the racemic gels were more thermally stable than either enantiomeric form—sometimes very significantly so. This is surprising as, normally, mixing enantiomers suppresses gelation [56,57,58]. Only in very rare cases have mixtures of enantiomers exhibited enhanced gelation [59,60,61,62,63,64,65,66].

Considering the results in more detail, the C4 amine (Figure 2, left) forms gels with L,L,L (or D,D,D) that have *T*_gel_ values of 51 °C. On addition of up to 20% of the other enantiomer, the *T*_gel_ value decreases, but once 30% of the other enantiomer is included, the *T*_gel_ value increases markedly, until reaching a maximum thermal stability for the racemic mixture. Similar (albeit smaller) trends were observed for C5 and C6. For C6, the optimal amine, the chirality of the dendron had the least effect on gel stability—suggesting this system is less sensitive to dendron chirality. For C7 (Figure 2, right, and C8), the initial addition of the enantiomer did not suppress thermal stability, but it did, once again, appear that a significant increase in *T*_gel_ was not achieved until >20% of the opposite enantiomer had been added.

In general, therefore, small amounts of the wrong enantiomer do not enhance gel stability, and may even suppress it, but once >20% is present, gelation is enhanced. This means each enantiomer must be present in sufficient quantity (>20%) for the gel to be reinforced. This suggests a mechanism in which L,L,L and D,D,D assemble individually. As such, we propose that homochiral recognition between molecular scale building blocks takes place, but that heterochiral interactions between the self-assembled homochiral fibres are then preferred and hence able to form the most effective gels. This is similar to the mechanism proposed by Žinić and co-workers in their landmark paper [59]. Supporting this view, the effects, as described above, are most pronounced with the amines that are least effective in supporting gelation in the first place (i.e., C4, C7, and C8)—these systems have the greatest ability to optimise overall fibre packing, which is relatively poorly mediated by the amine. For C6, where gelation is more effective in the first place, the optimisation offered by heterochiral fibre packing is much more limited.

On mixing D,L,L and L,D,D (see the supporting information, Figure S2), once again the racemic gels were more stable than those formed by individual enantiomers. For the mixing experiments, the *T*_gel_ plots were similar to those observed for L,L,L and D,D,D. We therefore propose that once again, for these dendrons, homochiral assembly into gel fibres is followed by heterochiral fibre-fibre interactions to form the most effective (i.e., racemic) gel.

**Figure 3:**
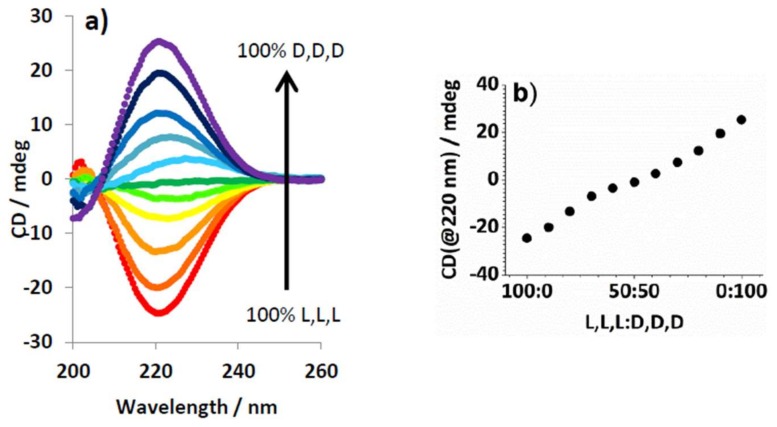
(**a**) CD spectra of mixtures of L,L,L and D,D,D (total concentration = 0.625 mM in), changing in 10% concentration increments in the presence of C8 (0.625 mM) from 100% LLL (red) to 100% LLL (purple); (**b**) Data extracted from graph (**a**) at 220 nm.

To further understand how the L,L,L/D,D,D ratio affected self-assembly, CD spectroscopy was used. Samples were made with dendron (0.625 mM total concentration), having varying ratios of enantiomers, and C8 (0.625 mM). C8 was selected because of the relatively large difference in thermal stability between enantiopure and racemic gels—the largest heterochiral preference. The absorbance of toluene in the wavelength region of interest (190–260 nm) meant samples were made in 95:5 methylcyclohexane/dioxane. This solvent still supported self-assembly to form gels at high enough concentration but produced optically transparent samples. The CD spectra (Figure 3a) indicated that enantiomeric gels have equal and opposite spectra. The change in ellipticity at 220 nm was plotted against the enantiomer ratio (Figure 3b) and shows an almost linear relationship. This clearly shows there is no “majority rules” effect, in which the excess of one enantiomer can enforce its preferred mode of organisation. This experiment would therefore agree with our suggestion that these enantiomers assemble into self-sorted homochiral structures, which then undergo heterochiral gel assembly—the CD signals of homochiral fibres would be expected to cancel one another out in a linear relationship as observed [31]. We attempted a similar experiment for D,L,L and L,D,D, but in this case the complexes formed had insufficient solubility in 95:5 methylcyclohexane/dioxane.

To further characterise the gels, VT-^1^H NMR spectroscopy in d_8_-toluene was used to detect the mobile components in the gel and hence infer what is included in the self-assembled “solid-like” gel network on the molecular scale [83,84,85,86]. Peptide gels (10 mM) formed with C8 (10 mM) were studied, with diphenylmethane (10 mM) included as a mobile internal standard so gelator peaks could be integrated and quantified (Figure S6). This method does not distinguish between L and D dendrons, as they have identical NMR spectra. From these experiments, the *T*_100%_ (temperature at which 100% of the dendron peptide gelator is in solution) was calculated for the racemic gel as 67 °C, compared with just 49 °C for the enantiopure gel. Molecular scale behaviour is therefore fully in agreement with the macroscopic observations—i.e., the racemic gel is significantly more thermally stable (Table 2) than the enantiopure analogue. The *T*_100%_ values are slightly higher than the macroscopic *T*_gel_ values (65 and 45 °C). This is expected [84], as the sample-spanning solid-like network becomes unable to support the gel somewhat before 100% of the gelator has fully dissolved. The [Insol]@*T*_gel_ values—the amount of gelator in the solid-like network at the gel–sol transition point—were also determined. For the enantiopure gel, [Insol]@*T*_gel_ was 2.8 mM, compared with only 0.5 mM for the racemic gel (Table 2). This indicates that much less solid-like network is required to underpin the racemic gel, i.e., self-assembly is more efficient. Macroscopic minimum gelation concentrations (MGCs) were determined (Table 2) and were in agreement with the molecular scale NMR study.

The same analysis was performed for L,D,D and D,L,L gels and their enantiomeric mixtures (Table 2, Figure S7). In this case, the system with C5 was analysed as it had the largest temperature difference between enantiopure and racemic gels. The *T*_100%_ values of 66 °C for the enantiopure gel and 77 °C for the racemic gel were in good agreement with the macroscopic *T*_gel_ values of 65 and 75 °C respectively, once again being slightly higher. The [Insol]@*T*_gel_ values were similar to one another, being 0.4 mM for the enantiopure gel and 0.7 mM for the racemic gel. Interestingly, however, the macroscopic MGCs were very different—6.0 mM for the enantiopure gel and 1.0 mM for the racemic gel. This would suggest that the enantiopure gelator has relatively high solubility, as a large total amount of gelator is required (6.0 mM) in order to establish a solid like fibre network, but only 0.4 mM is actually needed in the fibre network to underpin a gel. Based on this analysis, the racemic system, clearly has a much higher potential for aggregation into the solid-like state (i.e., lower solubility), as only 1.0 mM of gelator in total is required to establish a gel, with a minimum of 0.7 mM being needed in the solid-like network.

**Figure 4:**
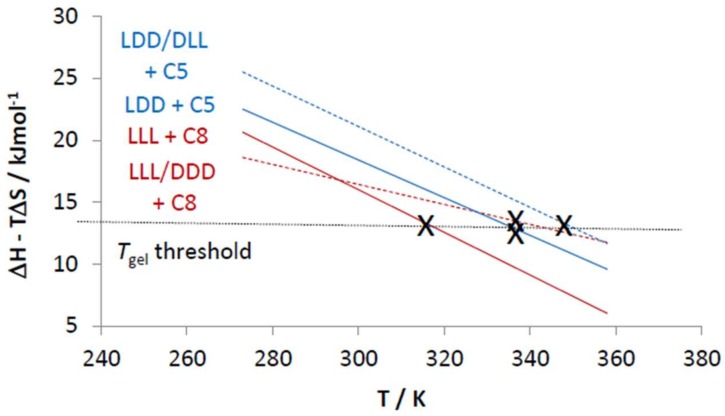
Plot of Δ*H* − TΔ*S* against temperature using the data from Table 3. X’s mark the experimental *T*_gel_ values. Enantiopure systems have full lines and racemic mixtures have dotted lines. This graph highlights the differences between systems and the apparent existence of a *T*_gel_ threshold.

We then carried out a van ’t Hoff analysis of the VT-NMR data to yield Δ*H*_diss_ and Δ*S*_diss_ values for the gel–sol transition (Table 3). Firstly, it should be noted that for all cases, there is an enthalpy–entropy balance [87,88]—if gelation is enthalpically more favoured (i.e., stronger interactions between gelators), then the increased order associated with the better packed network of the gel network makes it less entropically favoured. It is actually the balance between Δ*H*_diss_ and Δ*S*_diss_ that determines how thermally stable the gel really is (Δ*G* = Δ*H* − TΔ*S*). For L,L,L/D,D,D combined with C8, the racemic gel is more thermally stable because the gel has a much lower Δ*S*_diss_ value—i.e., the racemic gel is less entropically disfavoured, which more than compensates for it being less enthalpically favoured than the enantiopure gel. The lower enthalpy for gelation of the racemic mixture means less of the gelator is in the solid-like fibres at room temperature. However, the lower entropic cost of assembly for the racemic gel also means this racemic gel is less thermally sensitive (Δ*G* = Δ*H* − TΔ*S*) and the racemic gel therefore survives to higher temperatures than its enantiopure analogue. This can be demonstrated by plotting Δ*H* − TΔ*S* (i.e., Δ*G*) against temperature (Figure 4). The gradient of the line for the racemic mixture is much lower than that for the enantiopure system because of the lower entropy gain on conversion into a sol.

The behaviour of L,D,D and D,L,L with C5 is different to that for L,L,L and D,D,D with C8. In this case, the racemic gel is enthalpically more favoured. This is reflected in the fact that the racemic mixture has much lower solubility and more material is in the solid-like fibres. The enthalpically favoured packing in the racemic gel is more than able to offset the slightly increased entropic cost of gelation, and hence makes the gel more thermally stable. This can be seen by plotting Δ*H* − TΔ*S* against temperature (Figure 4)—although the gradients of the lines for the racemic mixture and the enantiopure system are similar, the fact that the racemic system starts off more enthalpically favoured means it does not disassemble until a higher temperature is reached.

Interestingly, all four systems investigated here appear to exhibit gel–sol transitions with very similar threshold values of Δ*H* − TΔ*S*, which suggests that the gels break down when the free energy associated with the gel–sol transition, and determined by the van ’t Hoff analysis, falls to a similar value in each case (ca 13 kJmol^−1^).

Clearly, changing structural features can significantly modify self-assembly and the thermodynamics of gelation. Furthermore, either enthalpy or entropy can play a dominant role in enhancing the thermal stabilities of these racemic gels, depending on the precise structural features. As such, even for closely related gels, it is important to realise that general sweeping conclusions about the thermodynamics of assembly cannot always easily be drawn.

**Figure 5:**
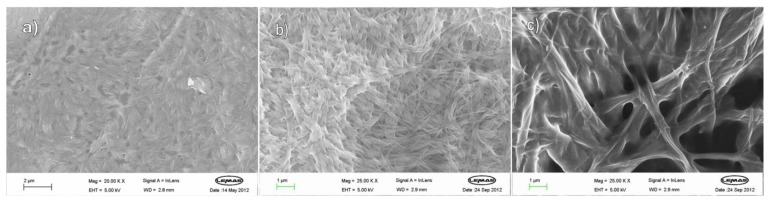
FEG-SEM images of xerogels formed by C8 and, (**a**) L,L,L; (**b**) D,D,D; (**c**) racemic mixture of L,L,L and D,D,D demonstrating differences in the nanoscale fibrillar morphology of the racemic gel.

FEG-SEM imaging was used to explore the nanostructures present in the xerogels (Figure 5). Although drying can have an effect on observed morphology, we reasoned that for this family of related gelators, as long as we kept sample preparation conditions the same in each case, then meaningful comparative conclusions could be drawn. Gels based on L,L,L and D,D,D with C8 appear, as expected, identical—composed of small fibres ca. 20 nm in width, which aggregate to form a continuous network. These fibres constitute nanoscale assemblies of molecular-scale fibrils. The racemic xerogel, on the other hand, has a very different network, being composed of thicker nanoscale fibres (ca. 50 nm), which then aggregate further to form even thicker, smoother fibres that are part of the continuous network. This would support the view that molecular-scale fibrils assemble very differently depending on whether the system is homochiral or heterochiral, and that this differential assembly on the nanoscale may underpin the enhanced thermal stability of the racemic gelator—in this case, heterochiral assembly yields the more thermally stale network. FEGSEM imaging of L,D,D and D,L,L in the presence of C4 led to similar conclusions (see Supplementary Material), with the enantiopure xerogels having networks of very thin fibres which were only just visible, while the racemic xerogel had a much more clearly visible network of fibres (ca. 20 nm), which aggregated even further to form even thicker fibres (ca. 100 nm). This supports the view that different heterochiral assembly of homochiral molecular-scale fibrils on the nanoscale underpins the enhanced performance of the racemic gel.

Interestingly, our previous work reported that when the same dendron acids were mixed with diamines in a 2:1 complex (rather than using monoamines in a 1:1 complex), they suppressed gelation when presented as a racemic mixture, rather than enhancing it [82]. The 1:1 complexes studied here can only exist in enantiomeric form, but if there are two peptide head units present in a 2:1 complex, the peptides they can either be both L,L,L, both D,D,D, or one L,L,L and one D,D,D. Our previous 2:1 system therefore had diastereomeric complexes present—we suggest that these diastereomeric complexes suppressed the self-assembly event, presumably as they could not pack so effectively. 

In summary, this new study reports that in this 1:1 system, a racemic mixture of dendrons is more effective than the enantiopure counterparts. This is an unusual observation and, depending on the precise structure of the system, can either be enthalpically or entropically driven.

### 2.4. Chiral Dendron and Chiral Amines

**Figure 6:**
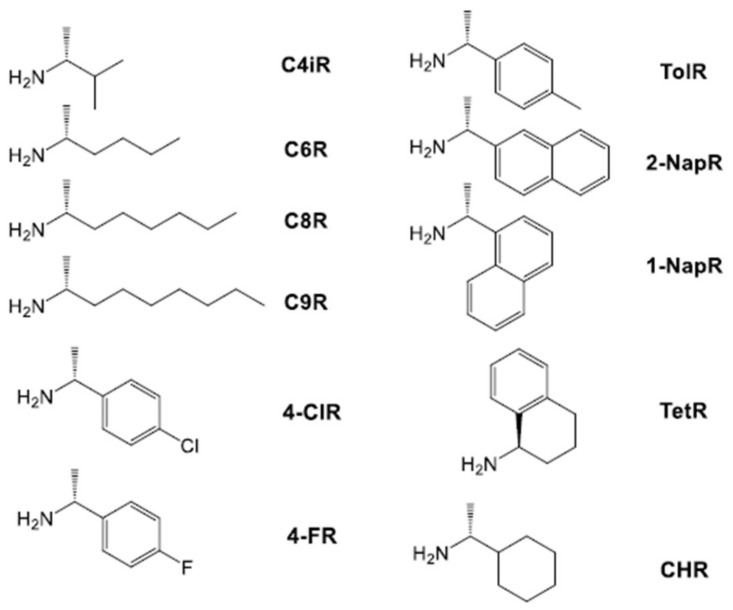
Chiral amines used to probe their directing effect on mixtures of peptide enantiomers.

Having studied the peptide dendrons with achiral amines, they were then tested with a collection of chiral amines (Figure 6).

**Figure 7:**
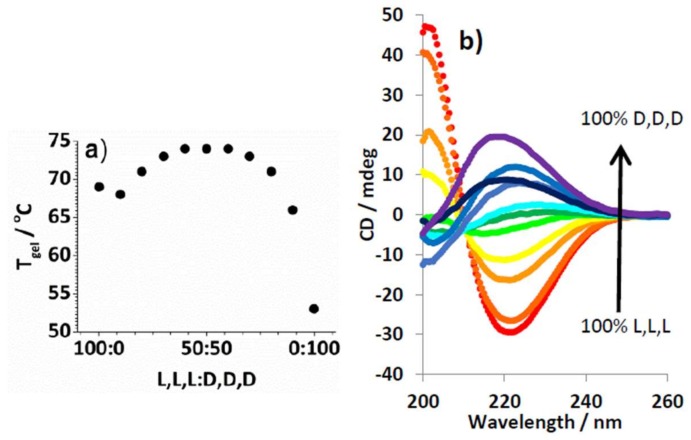
(**a**) Effect of mixing L,L,L and D,D,D (total combined concentration, 10 mM) on the *T*_gel_ value in the presence of C8R (10 mM); (**b**) CD spectra of mixtures of L,L,L and D,D,D (total concentration = 0.625 mM), changing in 10% concentration increments from 100% LLL (red) to 100% DDD (purple) in the presence of C8R (0.625 mM).

We had previously reported that L,L,L is capable of enantioselective gel assembly, for example favouring C6R over C6S [81]. It was therefore interesting to see how the mixture of enantiomeric peptides would perform when faced with a chiral amine (Figure 6a), as this provides insight into how multiple chiral components interact with one another. The first amine tested was C8R. From the *T*_gel_ values (Figure 7a), it is evident that C8R forms a more effective gel with L,L,L (69 °C) than D,D,D (52 °C)—a clear difference in gelation ability between these diastereomeric complexes. Interestingly, however, even relatively small amounts of L,L,L significantly improved the thermal stability of gels formed primarily with D,D,D—indeed once just 20% of L,L,L was present, the *T*_gel_ values were all fairly similar (ca. 70 °C), with the heterochiral L,L,L/D,D,D system being possibly slightly more effective.

CD spectroscopy with C8R (0.625 mM) and lysine dendron (total concentration 0.625 mM), with varying ratios of L,L,L to D,D,D in 95:5 methylcyclohexane/dioxane, indicated a clear change in CD spectrum (Figure 7b). Obviously, the spectra do not exhibit an enantiomeric relationship, as the complexes formed between chiral dendrons and a chiral amine are diastereomeric. The largest change in spectra occurs once small amounts of L,L,L have been added—in agreement with the macroscopic changes in *T*_gel_,. Indeed, it appears that a change in the nature of the spectrum occurs once 20% L,L,L is present, with the CD maximum shifting from ca. 218 nm to 221 nm, suggesting a switch to a packing mode which is becoming dominated by L,L,L rather than D,D,D. This would suggest that small amounts of L,L,L may direct overall assembly. This behaviour is quite different to that observed for the equivalent achiral amine (C8)—suggesting that the L,L,L dendron may exert some directing preferences onto the self-assembly/gelation event.

VT-NMR spectroscopy was then employed (Table 4, Figure S8), and a correlation between molecular-scale *T*_100%_ estimated by NMR and macroscopic *T*_gel_ values was again observed, with the *T*_100%_ values being slightly higher than the *T*_gel_ values. This confirms that on the molecular scale the L,L,L gel is more stable than D,D,D and that the mixed L,L,L/D,D,D system has a similar, or even slightly higher, thermal stability. Interestingly, L,L,L also has a lower MGC value than D,D,D or the mixed system, suggesting it does indeed has a greater driving force for assembly, as less is required for a gel to form. This is mirrored by the [Insol]@*T*_gel_ values which demonstrate that the L,L,L system can support a gel based on less solid-like network being present.

Thermodynamic analysis (Table 5) indicates that the D,D,D system (perhaps surprisingly) forms an enthalpically more favoured gel with C8R than L,L,L. However, the entropic cost of this more than offsets the enthalpic gain and means that gelation is less favoured. As such, L,L,L forms better gels with C8R for entropic reasons. Interestingly, the gel formed from a mixture of L,L,L and D,D,D has similar values to that formed with L,L,L alone, explaining why the two samples have similar *T*_gel_ and *T*_100%_ values. This provides further evidence suggesting that the complex formed between L,L,L and C8R is capable of directing the aggregation of the mixed L,L,L/D,D,D system in a manner reminiscent of the ‘majority rules’ mechanism [67,68,69,70,71,72,73,74].

**Figure 8:**
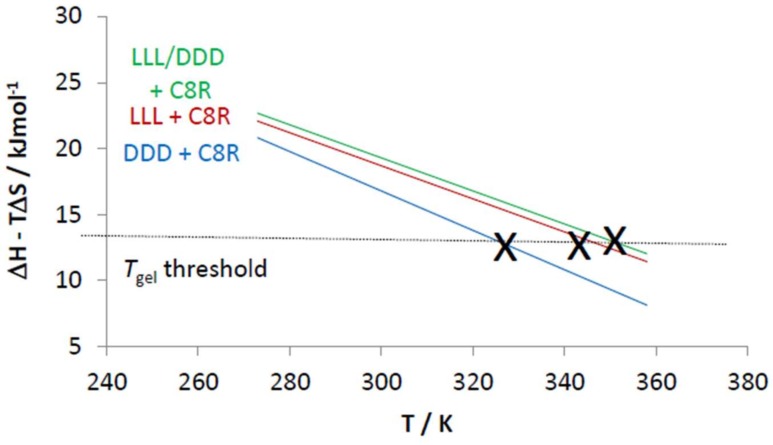
Plot of Δ*H* − TΔ*S* against temperature using the data from Table 5. X’s mark the experimental *T*_gel_ values reported in Table 4. This graph highlights the differences between systems and the apparent existence of a *T*_gel_ threshold.

Once again, plotting Δ*H* − TΔ*S* against temperature was informative (Figure 8), indicating that each gel undergoes a gel–sol transition as the free energy value of Δ*H* − TΔ*S* falls to a threshold level (ca. 13 kJmol^−1^). This demonstrates how the different thermodynamics derived from molecular-scale NMR studies control macroscopic gel thermal stability.

**Figure 9:**
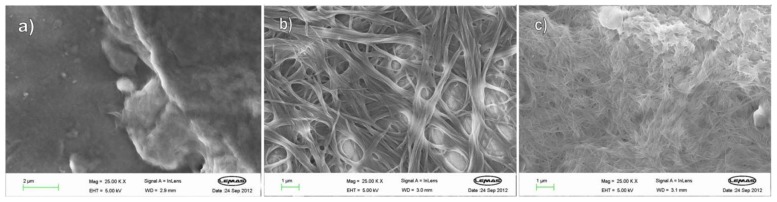
FEG-SEM images of xerogels formed by: (**a**) L,L,L and C8R; (**b**) D,D,D and C8R; (**c**) L,L,L + D,D,D and C8R.

FEG-SEM imaging of the xerogels formed by L,L,L and C8R (Figure 9) indicated very thin fibres (ca. 10–15 nm), barely visible even under high magnification. In contrast, D,D,D and C8R formed thicker fibres (ca. 200 nm)—it is evident that the molecular-scale thermodynamic differences are being expressed on the nanoscale and translated into the macroscopic performance. The mixed L,L,L and D,D,D sample consisted of fibres of ca. 50–100 nm, suggesting that, to some extent, L,L,L can impart its better network forming characteristics onto D,D,D, but also suggesting that L,L,L cannot totally dominate the nanoscale assembly event. This contrasts with the *T*_gel_ values for LLL + C8R and LLL/DDD + C8R, which were very similar, but is in agreement with the observed differences in MGC between L,L,L and the L,L,L/D,D,D hybrid.

A wide range of other chiral amines were then rapidly screened to determine if similar effects on *T*_gel_ were observed (see Supplementary Material for full data, Figures S3–S5). For aliphatic amines, the gels formed from either C4iR, C6R, or C9R demonstrated very similar trends to those formed with C8R, with L,L,L (and the L,L,L/D,D,D mix) forming more effective gels than D,D,D, and ca. 20% of L,L,L being sufficient to switch the apparent behaviour. Full data can be found in Supporting Information. With CHR, which has a cyclohexane ring, the chirality of the dendron appeared to have relatively little impact on *T*_gel_. This may reflect the significant difference in structure of this cyclic amine.

For aromatic benzylamines—4-MeR, 4-ClR, and 4-FR—in each case, L,L,L gave rise to more thermally stable gels. In most cases it was also clear that the gels formed from a 50:50 mix of enantiomeric dendrons showed somewhat enhanced thermal stability—similar to what was observed with achiral amines earlier.

More sterically demanding 1-NapR, 2-NapR, and TetR were then studied. The L,L,L dendron formed more effective gels than D,D,D with 1-NapR.^†^ Interestingly, however, increasing the ratio of L,L,L to D,D,D gradually increases the thermal stability, rather than maximising it after addition of only ca. 20%. TetR behaves in a very similar way. This might suggest that for these more hindered amines, the chiral L,L,L peptide cannot dictate its assembly preference as effectively, perhaps due to hindered packing. The resulting mixed gels therefore appear to behave more like simple mixtures of the individual enantiopure gels. For 2-NapR, as for CHR, there was limited impact of dendron chirality on thermal stability.

In summary, the presence of a chiral “*R*” amine has a pronounced effect on how the chirality of the lysine dendron controls gel formation. In all cases, L,L,L is equal to or better than D,D,D in terms of gelation ability. This demonstrates that molecular recognition pathways between the chiral dendrons direct the assembly. In some cases, relatively small amounts of L,L,L appear to impose a chiral preference onto D,D,D, suggesting chiral direction. However, in other systems, particularly where the amine is more hindered, directing effects appear to be absent.

## 3. Conclusions

This study reports the impact of chirality on the self-assembly of a two-component gel. Key observations are summarised in Table 6. For achiral amines, racemic mixtures form the best gels. The data are compatible with a model in which L,L,L and D,D,D (or D,L,L and L,D,D) form homochiral fibres with a preference for heterochiral packing. Thermodynamic studies demonstrated this can be either entropically or enthalpically driven, depending on the system being studied, and that it can be difficult to draw simple general conclusions, even within a structurally similar family of gelators.

Chiral amines with an *R* chiral centre always formed more stable gels with L,L,L than D,D,D—suggesting this diasteromer is better able to self-assemble. It appeared that in some cases, L,L,L can imposes its chiral preference onto D,D,D and directs the overall assembly event, however, this was less apparent if the amine was sterically hindered. Interestingly, all gels studied in this paper appeared to break down when Δ*H* − TΔ*S* fell to a threshold value (ca. 13 kJmol^−1^).

In summary, this paper provides unique insights into chiral gels and reports some unusual phenomena, demonstrating that self-assembled gels are responsive to molecular scale chiral information programmed into them, which can operate over a variety of length scales, from molecular- and nanoscale all the way up to macroscale. Given the importance of chiral recognition pathways in biological and materials science, we suggest that these results will be of significance in helping understand how complex multi-component chiral soft matter systems can be organised.

## 4. Materials and Methods 

### 4.1. Synthesis and Characterisation

The four dendritic peptides based on G2-Lys used in this study (L,L,L, D,D,D, D,L,L and L,D,D) were synthesised using methods disclosed in reference [82] and all spectroscopic data were in full agreement with those previously reported. 

### 4.2. Experimental Methods

#### 4.2.1. Gel Formation 

Stock solutions of the dendron and the amine were made. Amounts of these stock solutions were added to a 2 mL sample vial with any excess toluene if required using a Gilson pipette. Often a gel was formed instantly upon mixing. This sample was then heated with a heat gun until a homogeneous, clear solution was formed. The sample was then left at room temperature overnight to cool, over which time the sample gelates. This process was undertaken to ensure the gels formed were homogeneous. When mixtures of amines were used in a gel, these stock solutions were mixed first, before the other component was added. This was to ensure mixing with each different amine occurred at the same time.

#### 4.2.2. *T*_gel_ Measurements 

Gel samples were placed in a thermostatted oil bath in a 2 mL sample vial, and heated at a rate no faster than 0.5 °C/min. As the temperature was increased, the gel was removed from the oil bath and turned upside down. The temperature at which the gel could no longer support itself against gravity—when the gel collapses—was taken as the *T*_gel_ of the sample. All *T*_gel_ values were repeated at least once.

#### 4.2.3. Field Emission Gun Scanning Electron Microscopy (FEG-SEM) 

Once a gel had set, a small amount was removed with a spatula and spread thinly onto an aluminium SEM stub. This was allowed to air dry in a desiccator to leave the xerogel. This xerogel was then coated with a layer of Pt/Pd and viewed under the microscope. 

#### 4.2.4. Circular Dichroism (CD) 

An amount of methylcyclohexane (spectrophotometric grade) was pipetted into a 2 mL sample vial. An amount of amine stock solution (in methylcyclohexane) was then added. This was followed by addition of dendron stock solution (in dioxane). This sample was then heated until a clear homogeneous solution was formed. This was left to cool overnight so any aggregation can take place. For analysis the samples were gently pipetted into the CD cuvette and analysed in the spectrometer. 

#### 4.2.5. NMR Studies 

Stock solutions of amine, dendron and internal standard diphenylmethane (DPM) in toluene-*d*_8_ were mixed in an NMR tube. The amines were always added to the tube before the dendron so mixing with all occurred at the same time. The mixture was heated with a heat gun until a clear, free flowing solution was formed. Following this, the sample was left to cool and equilibrate overnight, over which time gelation occurred. The ^1^H NMR spectra of the sample was recorded at 5 °C intervals as the temperature increased from 25–85 °C. The integration of the relevant peaks were recorded at each temperature and converted to concentration by comparison to the internal standard. The van ’t Hoff plots were produced using Equation (1), using the method proposed in previous work reference [84]. The gradient of the plots is equal to −Δ*H_diss_*/R and the intercept equal to Δ*S_diss_*/R. The calculated values of Δ*H_diss_* and Δ*S_diss_* were used to predict the concentration of solubilized gelator at each 5 °C temperature interval. This was compared to the experimentally measured results to provide verification of this model.

(1) $$\ln\left(Sol\right)=-\frac{\Delta H_{diss}}{RT}+\frac{\Delta S_{diss}}{R}$$

## Figures and Tables

**Table 1:** *T*_gel_ values (°C) for peptide dendrons denoted by their chirality, with different non-chiral amines (C4–C8) in toluene. [Dendron] and [amine] both 10 mM.

Amine	l,l,l	d,d,d	d,l,l	l,d,d
C4	51	51	71	71
C5	64	64	65	65
C6	70	70	70	70
C7	53	53	69	69
C8	45	45	54	54

**Table 2:** Comparison of data from VT NMR experiments with dendrons/amines and macroscopically observed data for gels with 10 mM amine and 10 mM total dendron concentration in d_8_-toluene.

Dendron	Amine	*T*_100%_^a^/°C	*T*_gel_^b^/°C	[Insol]@*T*_gel_^c^/mM	MGC ^d^/mM
L,L,L	C8	49	45	2.8	4.0
L,L,L + D,D,D	C8	67	65	0.5	2.8
L,D,D	C5	66	65	0.4	6.0
L,D,D + D,L,L	C5	77	75	0.7	1.0

^a^ *T*_100%_ is the temperature at which 100% of the gelator is in the liquid-like phase by NMR; ^b^ *T*_gel_ is the macroscopically observed gel-sol transition; ^c^ [Insol]@*T*_gel_ is the concentration of gelator in solid-like state as assessed by NMR at the *T*_gel_ value; ^d^ MGC is the minimum gelation concentration at room temperature, i.e., the minimum total amount of gelator required to form a gel.

**Table 3:** Thermodynamic data derived from van ’t Hoff plots as determined using NMR methods.

Peptide Dendron	Amine.	Δ*H*_diss_^a^/kJmol^−1^	Δ*S*_diss_^a^/Jmol^−1^ K^−1^
L,L,L	C8	67.6	172
L,L,L + D,D,D	C8	41.7	84.6
L,D,D	C5	64.0	152
L,D,D + D,L,L	C5	70.0	163

^a^ Thermodynamic terms refer to the dissociation process from gel to sol.

**Table 4:** Comparison of data calculated from VT NMR experiments with peptides and C8R and macroscopically observed data for gels with 10 mM amine and 10 mM total peptide dendron concentration in d_8_-toluene.

Peptide Dendron	*T*_100%_^a^/°C	*T*_gel_^b^/°C	[Insol]@*T*_gel_^c^/mM	MGC ^d^/mM
L,L,L	72	69	0.5	0.6
L,L,L + D,D,D	75	74	1.2	1.4
D,D,D	55	53	1.3	1.6

**Table 5:** Thermodynamic data derived from van ’t Hoff plots with C8R and peptide dendrons as determined by NMR methods.

Peptide Dendron	Δ*H*_diss_/kJmol^−1^	Δ*S*_diss_/Jmol^−1^ K^−1^
L,L,L	56.2	125
L,L,L + D,D,D	56.8	125
D,D,D	61.5	149

**Table 6:** Summary of main observations for the thermal stability of gels based on G2-Lys.

Amines	Thermal Stability	Thermal Stability of Peptide Mixtures
Achiral Amines	L,L,L = D,D,D	Racemic Mixture Best
Chiral ‘R’ Amines	L,L,L > D,D,D	L,L,L ~ Racemic Mix >> D,D,D

## Supplementary Materials

The following are available online at http://www.mdpi.com/2310-2861/4/2/31/s1, Figure S1: Effect of enantiomeric mixing on *T*_gel_, as measured by tube inversion. Concentration of amine is 10 mM, and total concentration of dendron (D,D,D + L,L,L) is also 10 mM. Figure S2: Effect of enantiomeric mixing on *T*_gel_, as measured by tube inversion. Concentration of amine is 10 mM, and total concentration of dendron (D,L,L + L,D,D) is also 10 mM. Figure S3: Effect of mixing L,L,L and D,D,D (total combined concentration 10 mM) on the *T*_gel_ value in the presence of different acyclic chiral amines. Figure S4: Effect of mixing L,L,L and D,D,D (total combined concentration 10 mM) on the *T*_gel_ value in the presence of different cyclic chiral amines. Figure S5: Effect of mixing L,L,L and D,D,D (total combined concentration, 10 mM) on the *T*_gel_ value in the presence of different hindered chiral amines (10 mM). Figure S6: Concentration of lysine dendron visible in gels of L,L,L-G2Lys, or l,l,l-G2Lys and d,d,d-G2Lys with C8 as temperature increases (solvent: toluene-*d*_8_). Van ‘t Hoff plots of gels formed from l,l,l-G2Lys or l,l,l-G2Lys and d,d,d-G2Lys with C8. Figure S7: Concentration of lysine dendron visible in gels of l,d,d-G2Lys, or l,d,d-G2Lys and d,l,l-G2Lys with C5 as temperature increases (solvent: toluene-*d*_8_). Van ’t Hoff plots of gels formed from l,d,d-G2Lys or l,d,d-G2Lys and d,l,l-G2Lys with C5. Figure S8: Concentration of lysine dendron visible in gels of l,l,l-G2Lys, or d,d,d-G2Lys, or l,l,l-G2Lys and d,d,d-G2Lys with C8R as temperature increases (solvent: toluene-*d*_8_). Van ’t Hoff plots of gels formed from l,l,l-G2Lys or d,d,d-G2Lys or l,l,l-G2Lys and d,d,d-G2Lys with C8R.
