# Supplementary material for: Chiral Assembly Preferences and Directing Effects in Supramolecular Two-Component Organogels
Source: Gels. 2018 Mar 29;4(2):31. doi: 10.3390/gels4020031 (PMC6209267; doi:10.3390/gels4020031)
Supplement: Supplementary file 1 [file gels-04-00031-s001.pdf]

Enhanced thermal stability and chiral directing effects in heterochiral self-assembled gels

William Edwards[a] and David K. Smith*[,a]

SUPPLEMENTARY INFORMATION

Contents

1 Thermal Stability Data

2 Variable Temperature NMR Spectroscopy Data

1. Thermal Stability

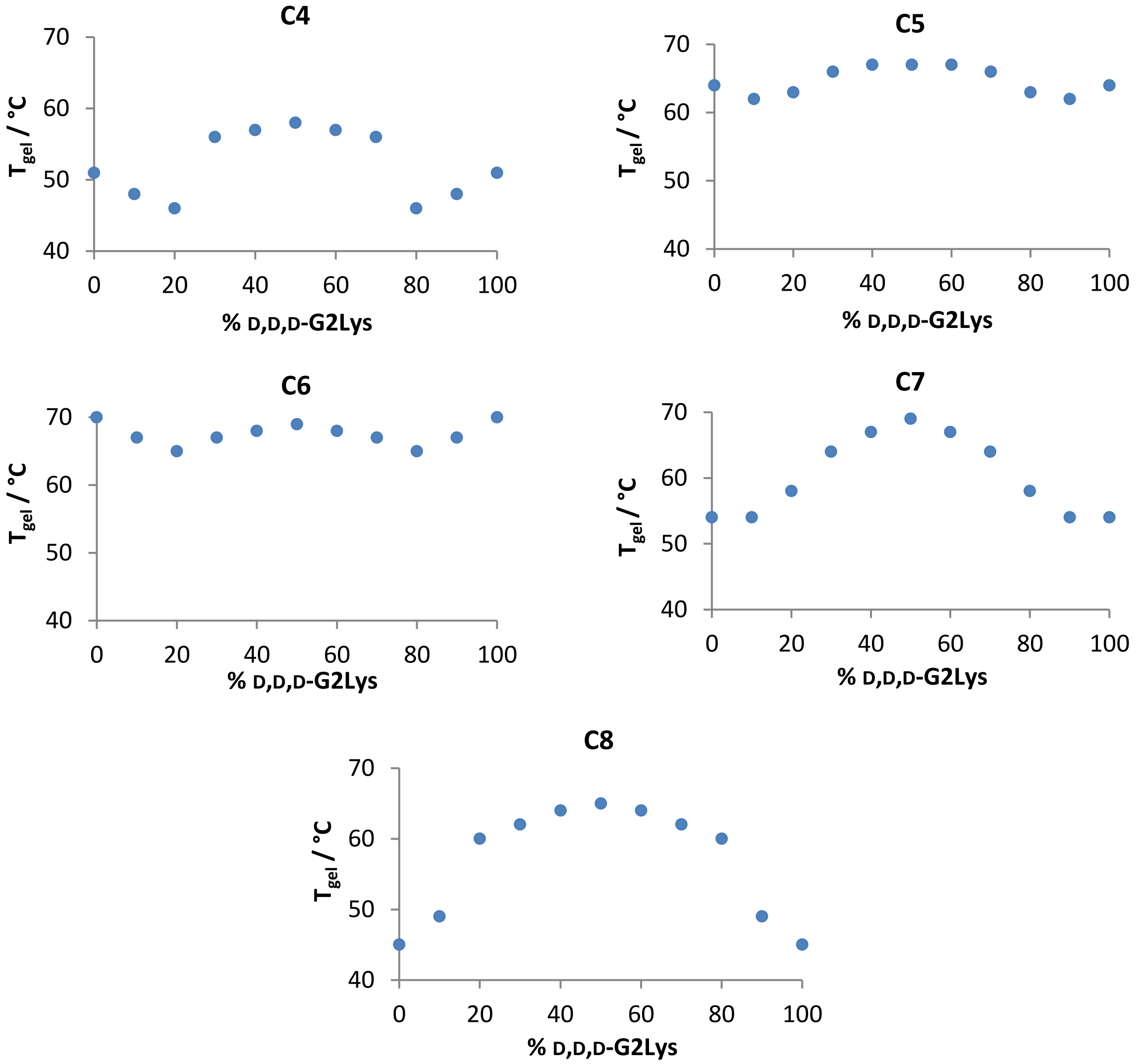

Figure S1. Effect of enantiomeric mixing on T_{gel}, as measured by tube inversion. Concentration of amine is 10 mM, and total concentration of dendron (D,D,D+L,L,L) is also 10 mM.

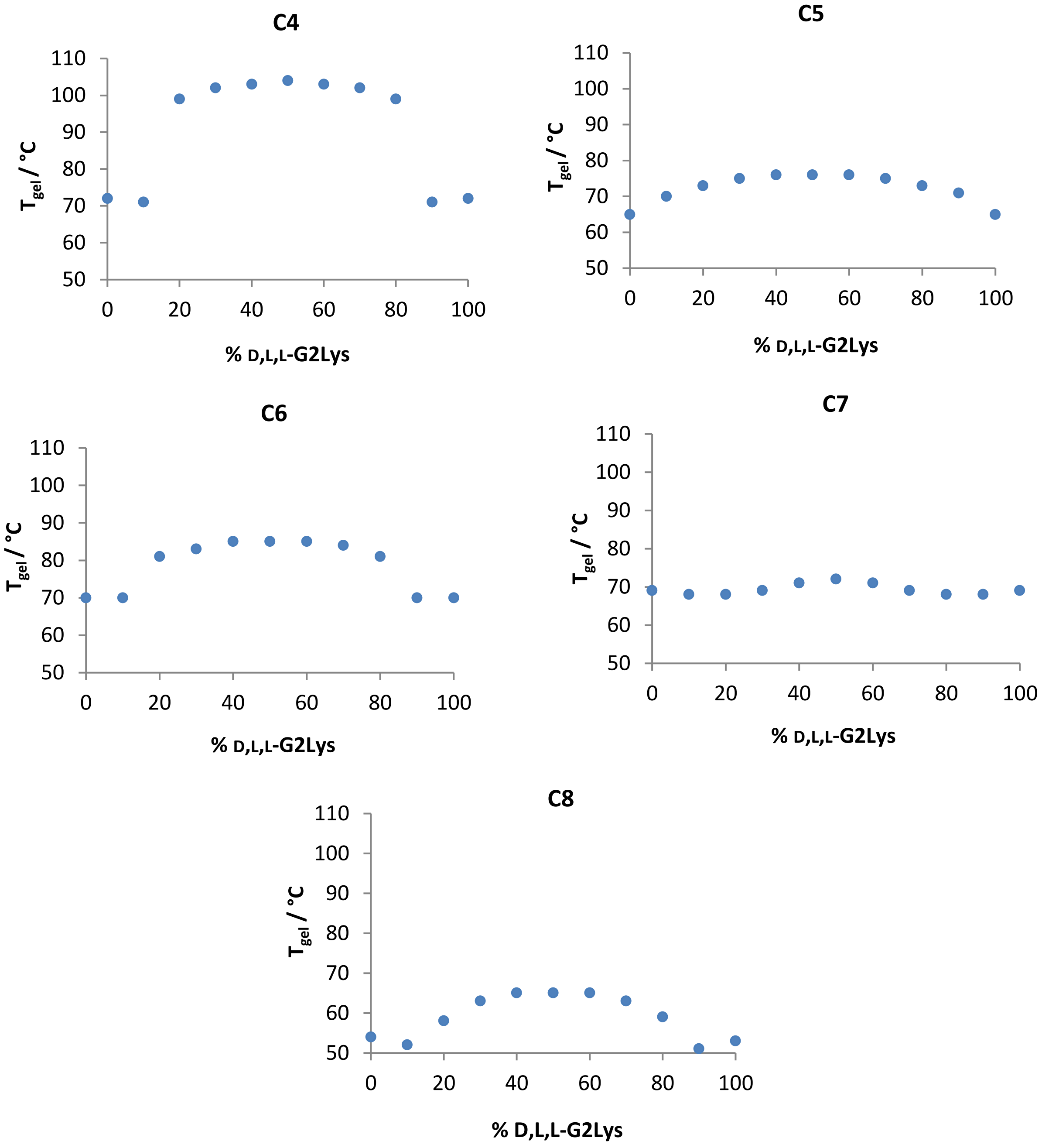

Figure S2. Effect of enantiomeric mixing on T_{gel}, as measured by tube inversion. Concentration of amine is 10 mM, and total concentration of dendron (D,L,L+L,D,D) is also 10 mM

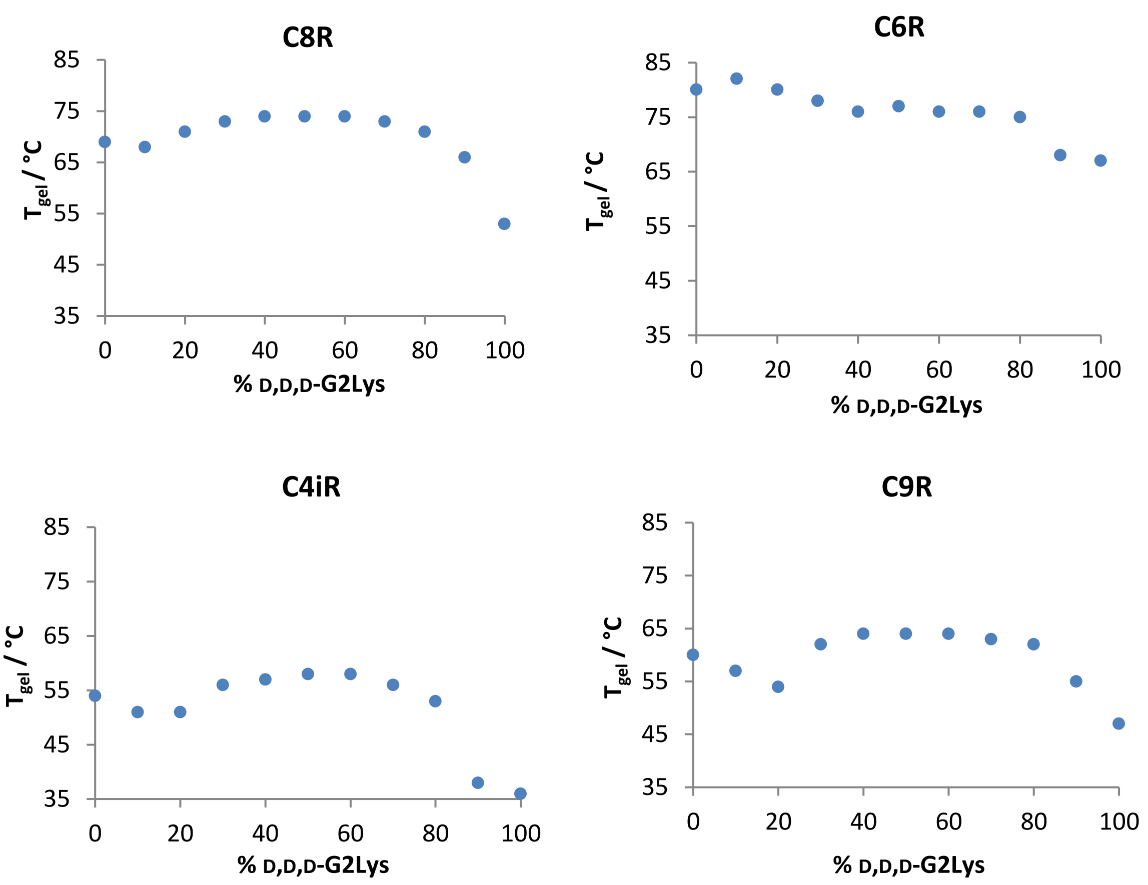

Figure S3. Effect of mixing L,L,L and D,D,D (total combined concentration 10 mM) on the T_{gel} value in the presence of different acyclic chiral amines.

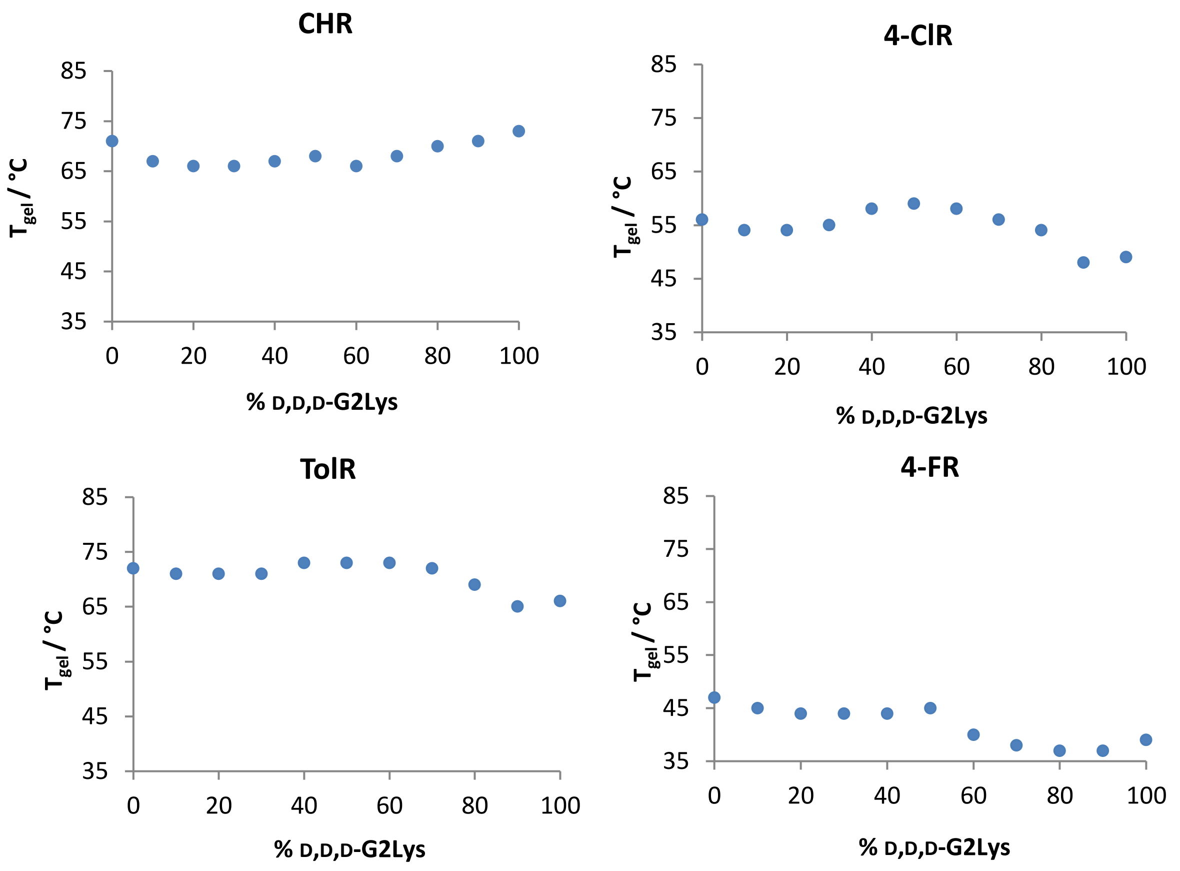

Figure S4. Effect of mixing L,L,L and D,D,D (total combined concentration 10 mM) on the T_{gel} value in the presence of different cyclic chiral amines.

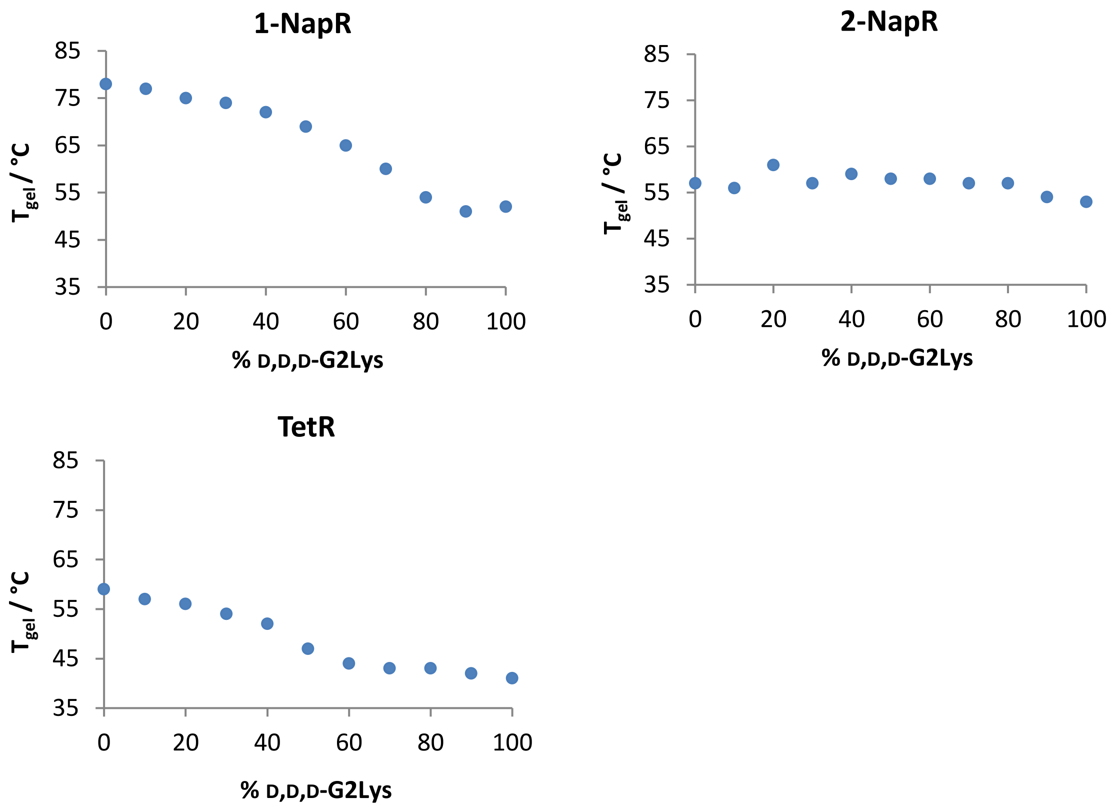

Figure S5. Effect of mixing L,L,L and D,D,D (total combined concentration, 10 mM) on the T_{gel} value in the presence of different hindered chiral amines (10 mM).

2. Variable Temperature NMR Spectroscopy Data

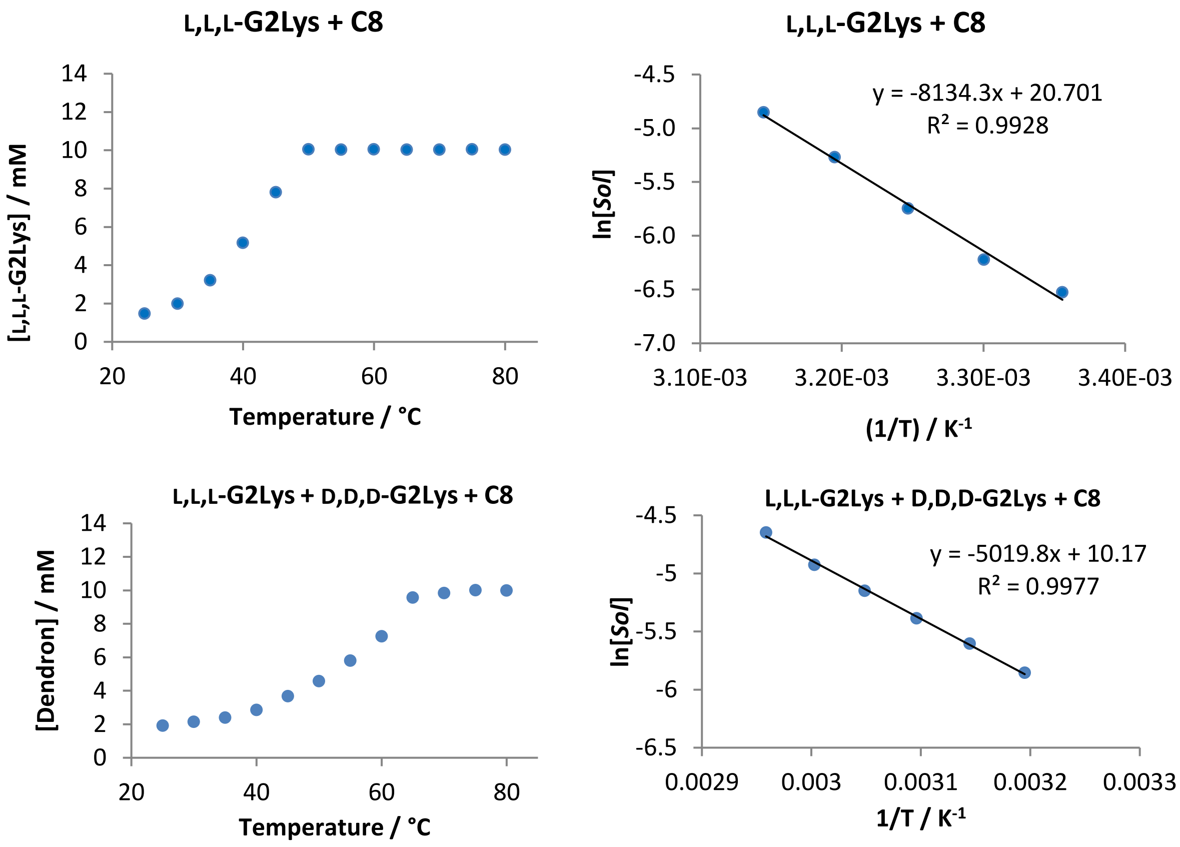

Figure S6. Concentration of lysine dendron visible in gels of **L,L,L-G2Lys,** or **L,L,L-G2Lys** and **D,D,D-G2Lys** with **C8** as temperature increases (solvent: toluene-d_8). Van 't Hoff plots of gels formed from **L,L,L-G2Lys** or **L,L,L-G2Lys** and **D,D,D-G2Lys** with **C8.**

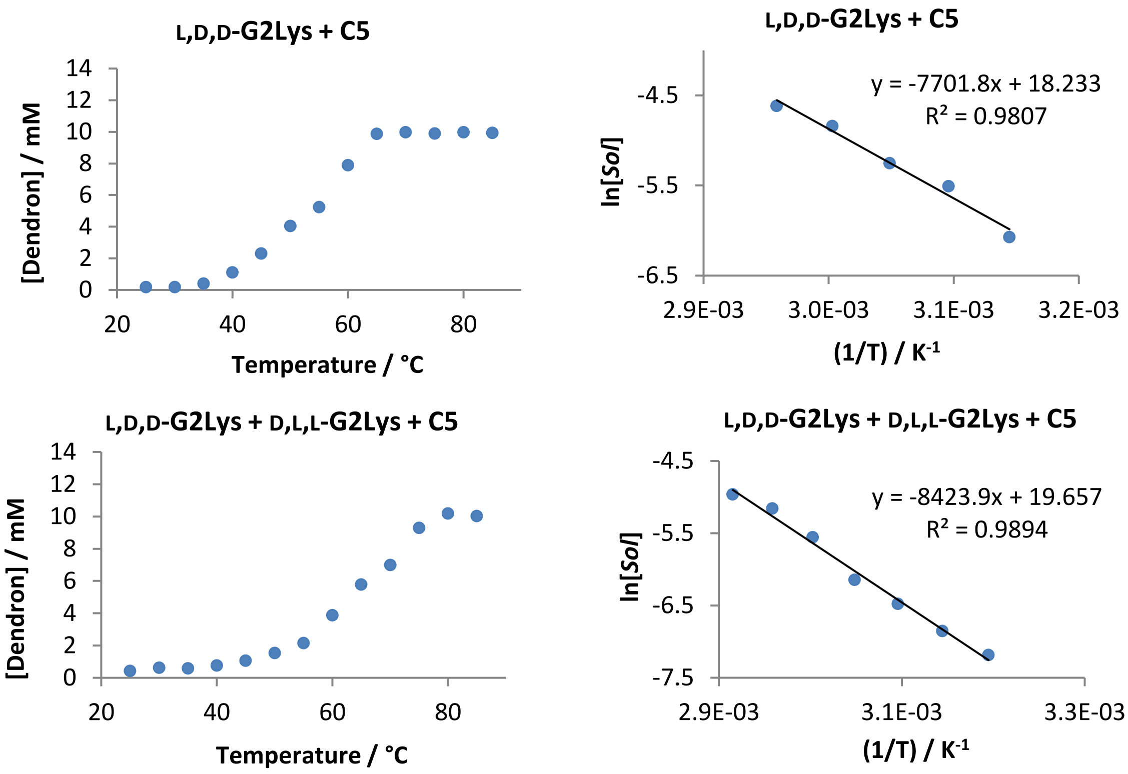

Figure S7. Concentration of lysine dendron visible in gels of **L,D,D-G2Lys,** or **L,D,D-G2Lys** and **D,L,L-G2Lys** with **C5** as temperature increases (solvent: toluene-d_8). Van 't Hoff plots of gels formed from **L,D,D-G2Lys** or **L,D,D-G2Lys** and **D,L,L-G2Lys** with **C5.**

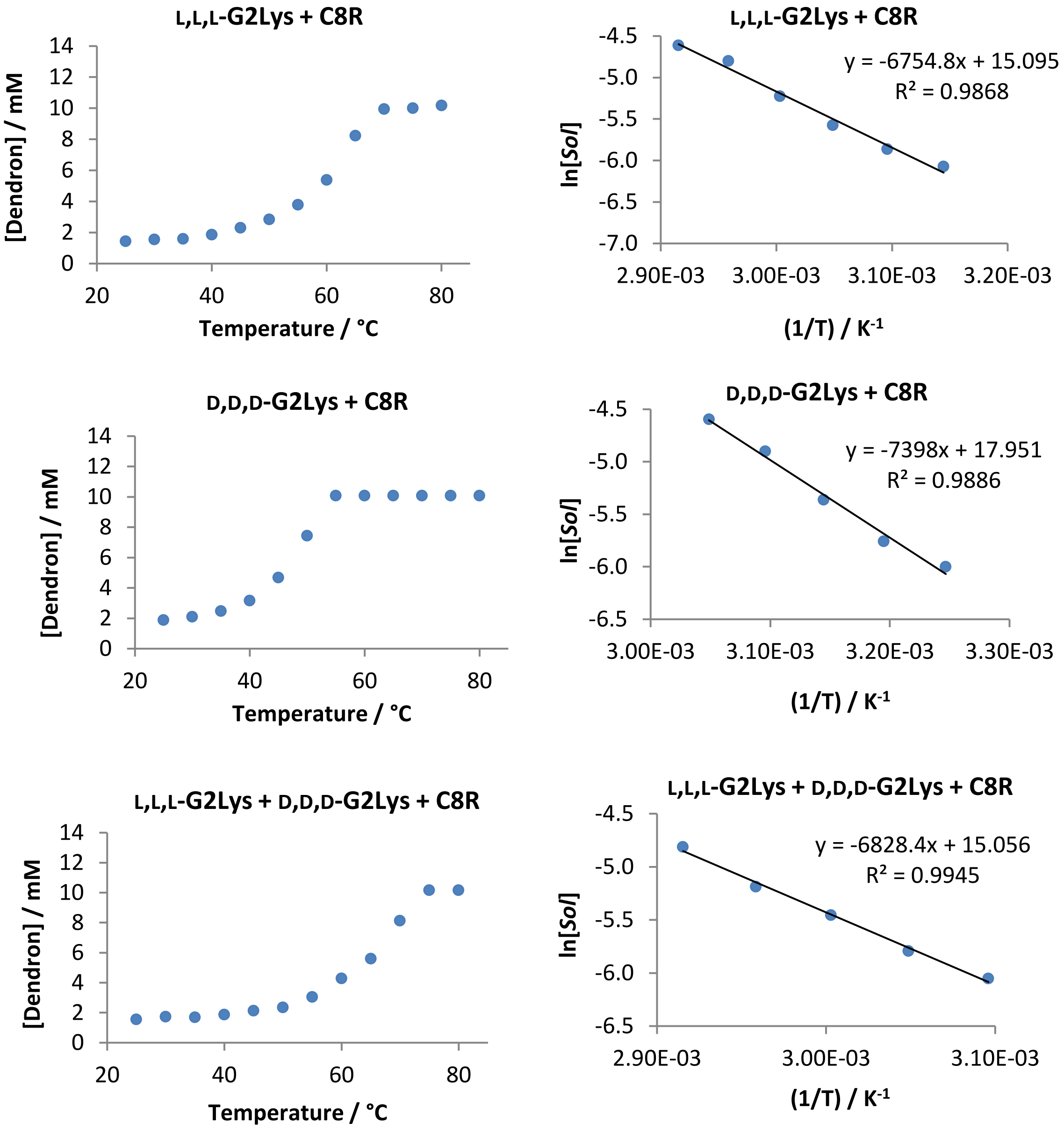

Figure S8. Concentration of lysine dendron visible in gels of **L,L,L-G2Lys,** or **D,D,D-G2Lys**, or **L,L,L-G2Lys** and **D,D,D-G2Lys** with **C8R** as temperature increases (solvent: toluene-d_8). Van 't Hoff plots of gels formed from **L,L,L-G2Lys** or **D,D,D-G2Lys** or **L,L,L-G2Lys** and **D,D,D-G2Lys** with **C8R.**
